# Exponential Families with External Parameters

**DOI:** 10.3390/e24050698

**Published:** 2022-05-14

**Authors:** Marco Favretti

**Affiliations:** Dipartimento di Matematica Tullio Levi-Civita, Università degli Studi di Padova, 35131 Padova, Italy; favretti@math.unipd.it

**Keywords:** exponential family, Fisher metric, Maximum Entropy principle, Ehresmann connection, thermodynamic length, Fokker-Planck equation, generalized Phytagorean theorem

## Abstract

In this paper we introduce a class of statistical models consisting of exponential families depending on additional parameters, called external parameters. The main source for these statistical models resides in the Maximum Entropy framework where we have thermal parameters, corresponding to the natural parameters of an exponential family, and mechanical parameters, here called external parameters. In the first part we we study the geometry of these models introducing a fibration of parameter space over external parameters. In the second part we investigate a class of evolution problems driven by a Fokker-Planck equation whose stationary distribution is an exponential family with external parameters. We discuss applications of these statistical models to thermodynamic length and isentropic evolution of thermodynamic systems and to a problem in the dynamic of quantitative traits in genetics.

## 1. Introduction

This work is a first attempt to study the geometrical properties and potential applications of a class of statistical models consisting of exponential families depending on additional parameters, called external parameters. The main source for these statistical models comes from the application of E.T. Jaynes Maximum Entropy framework [1] to thermodynamical systems, where we can identify in a natural way thermal parameters (corresponding to natural parameters in an exponential family) and mechanical parameters, here called external parameters. While the construction of equilibrium Statistical Mechanics from the Maximum Entropy principle is a well established domain of science, little attention is paid in the literature to the intrinsic geometrical structure of these statistical models. Given the widespread application of Maximum Entropy principle to disparate fields of science, it is reasonable to assume that a closer scrutiny of these models can pave the way to further applications outside statistical thermodynamics.

Here is the plan of the paper: in Section 2 we recall the definitions of regular statistical model and of exponential family. The main point is that we are dealing with a finite dimensional Riemannian manifold with respect to the Fisher metric. In Section 3 we introduce the exponential families with external parameters, we state the conditions that render them a regular statistical model and we compute the Fisher metric. The additional geometrical structure that we get with these exponential families is a fibration over the space of external parameters *U* in the sense that for every fixed u∈U the fiber is a standard exponential family. The notion of Eheresmann connection on a fibered bundle and of parallel transport is recalled in Section 4. In Section 5 we outline some applications of these parameterized exponential families: we give a formula for the thermodynamic length of a process described by a path in both natural and external parameters and we give conditions for the isentropic evolution of the system. Section 6 is motivated by a model problem in quantitative genetics (briefly recalled in Appendix A) where the dynamics of the system is given by a Fokker-Planck equation with gradient drift and the equilibrium or stationary distribution is an exponential family with external parameters. We recast the dynamic approximation procedure exposed in [2,3,4] in the framework of exponential family with external parameters and we give a generalization of the ODE that drives the approximating dynamics. We think that the consideration from the present point of view of the problem exposed in [2,3,4] may shed light on some still poorly understood aspects of the model.

### Exponential Families in Statistical Thermodynamics

To help locate the contribution of the present paper in the scientific literature we briefly review and compare some of the geometrical approaches to statistical mechanics that are most relevant for our argument. A line of research initiated by the influential papers of Wheinhold [5] and Ruppeiner [6] investigates the Riemannian metric structure on parameter space related to the Boltzmann-Gibbs canonical distribution. This Riemannian metric is the one defined by the Hessian matrix of the free energy ψ=logZ (which coincides with the Fisher metric) with respect to the canonical parameters or by its inverse which is the Hessian of the entropy *S*, related to ψ by the Legendre transform. The Levi-Civita connection with respect to this metric allows to define the Riemannian curvature tensor and its sectional and scalar curvature. For a two-dimensional parameter space the divergence of the scalar curvature is a signal of the existence of a phase transition in the underlying physical system. This theory has been applied to Ising and Potts lattice system, to the ideal and Van der Waals gas and to black hole thermodynamics (see e.g., [7,8,9,10]). However in dimension grater of two the scalar curvature has a less stringent role and care must be taken in the interpretation of the results.

In this work we also start from the Boltzmann-Gibbs distribution but we stress the different role of natural or thermal parameters θ, which occur linearly in (15), and external parameters *u* which may enter nonlinearly in the Boltzmann-Gibbs distribution. In particular we are interested in using the external parameters as control parameters on the evolution of the system. The related geometrical framework exposed in Section 4 adopts the connection and curvature associated to the Ehresmann connection on the fibration locally described by (θ,u)→u, which is fit for describing the isentropic evolution of the system or the dependence of the work control protocol on the global geometric structure i.e., the holonomy of the path of the external control space.

A second line of research relating information geometry and statistical thermodynamics concerns the notion of thermodynamic length (see [11,12,13]), which is important in the design of optimal driving protocols for the non-equilibrium evolution of (small) thermodynamic systems, see [14,15], both for classical and quantum descriptions. In this work (see Section 5.1) we investigate the notion of thermodynamic length using our geometric framework and we give a formula for for thermodynamic length that highlights the contribution of natural and external (controlled) parameters.

For the sake of completeness we cite the statistical models introduced by J. Naudits (see [16,17]) called generalized exponential families and *q*-exponential families by Amari-Ohara, [18]. In these models the exponential function is generalized by introducing the so-called *q*-deformed exponential. In practice one considers simultaneously two elements of an exponential family, the second one is called escort distribution. These deformed exponential families are useful for describing Tsallis thermostatistics [19] which gives a more accurate description for thermodynamic systems where the extensivity of the classical definition of entropy notion is defied. However this highly debated topic is not relevant for the present work.

This paper is a first attempt to study the exponential families with external parameters using geometrical tools. Even if we were inspired by the Maximum Entropy formalism our result are completely general. In particular we investigated the case where the family (with respect to the the natural and external parameters) is a regular statistical models. This is only a first step in the analysis of these parameterized models; a further step would be in the direction of singular (in opposition to regular) statistical models (see [20]) a domain where there is nowadays an increasing attention in the information geometry community. A drawback of this work is that most of the results are presented in a coordinate-dependent way and have a local character. We hope to resolve these issues in a subsequent work. Some of the results presented here were introduced in a less refined form in [21].

## 2. Statistical Models and Exponential Families

Before introducing their generalization in Section 3 below, we recall the definitions of regular statistical model and of exponential family (see [22,23,24]). Let (X,B,dx) be a probability space where *X* may be a discrete or continuous set. We stipulate that in case of a discrete set the integrals over *X* with respect to the measure dx are substituted by sum symbols. Let

$$P\left(X\right)=\left\{p:X\to \left[0,+\infty \right),\;p\left(x\right)\geq 0,\int_{X}pdx=1\right\}\subset L_{1}\left(X\right)$$

be the infinite dimensional space of probability densities over *X*. Let $Z\subset R^{d}$ be the open set of the parameters, f:Z⟶P(X) be a given smooth map and consider the subset of P(X)

S={p=f(z):z∈Z}⊂P(X).

To avoid technicalities, we stipulate that the support of *p*, i.e., the set where p>0 is the same for all p∈S and that it coincides with *X*. We now state the conditions under which S is a regular *d*-dimensional statistical model (see [22,24,25]).

**Definition** **1** (Regular statistical model)**.** *S is a regular statistical model if the following conditions are satisfied:*

*1*. *(injectivity) the map f:Z⟶S, z↦f(z)=p(z) is one to one,*
*2*. *(regularity) the d functions defined on X*
  $$p_{i}\left(x;z\right)=\frac{\partial p}{\partial z_{i}}\left(x;z\right),\;i=1,\ldots ,d$$
  *are linearly independent as functions on X for every z∈Z.*

A statistical model which is not regular is called singular (see [20] for a comprehensive discussion on singular models). If condition 1. hold the model is called identifiable, otherwise it is called unidentifiable. If condition 2. fail the main consequence is that the Fisher metric (22) is only positive semidefinite because condition (23) fail. Many statistical model e.g., Boltzmann machines, Bayes networks, hidden Markov models are singular. Note that for a regular statistical model the inverse φ of the map *f*, φ(p)=z defines a global coordinate system for S.

To check regularity condition 2. it is convenient to introduce the so called log-likelihood l=lnp of *p* and the score base

(1) $$l_{i}\left(x;z\right)=\frac{\partial l}{\partial z_{i}}=\frac{\partial \ln p}{\partial z_{i}}=\frac{1}{p}p_{i}\left(x;z\right).$$

Since $l_{i}$ and $p_{i}$ are proportional, the regularity condition 2. holds if and only if the elements of the score base are linearly independent on *X*.

### Exponential Family

Foundamental examples of statistical models are the exponential families. Let us introduce *a* observable functions $h:X\to R^{a},\;h=\left(h_{1},\ldots ,h_{a}\right)$ and suppose that the a+1 functions

(2) $$h_{1}\left(x\right),\ldots ,h_{a}\left(x\right),\;1,$$

are linearly independent as functions over *X*, where 1 denotes the constant function over *X*. Moreover, let k=k(x) be a function defined on *X* and let us introduce the *free energy* $\psi :\Theta \subset R^{a}⟶R$, ψ=ψ(θ) as (here θ·h denotes the scalar product in $R^{a}$)

(3) $$\psi (\theta )=\ln\int_{X}e^{\theta \cdot h(x)+k(x)}dx$$

where the parameter space Θ is the subset of $R^{a}$ where $e^{\psi }\left(\theta \right)<+\infty$. The *a* real numbers θ are called *natural* parameters. It is known that the set Θ is open and convex in θ and that ψ is a convex function in the θ variable (see [23,26]).

The following subset of the infinite dimensional space P(X)

(4) $$E=\{\;p\left(x;\theta \right)=e^{\theta \cdot h(x)-\psi (\theta )+k(x)},\;\theta \in \Theta \}\subset P\left(X\right).$$

is called exponential family. We show that E is an *a*-dimensional regular statistical model. For p∈E we have

(5) l=lnp=θ·h(x)−ψ(θ)+k(x)

therefore the injectivity condition 1. above holds if and only if for all $\theta ,\theta^{\prime }\in \Theta$

(6) $$\left(\theta -\theta^{\prime }\right)\cdot h\left(x\right)+1\left(\psi \left(\theta^{\prime }\right)-\psi \left(\theta \right)\right)=0\;\forall x\in X\;\Rightarrow \;\theta =\theta^{\prime }$$

holds and this is true by the independence condition (2) above. To check regularity condition 2 above, we compute the elements of the score base. They are (here we use the shorthand notation $\partial_{i}f=\partial f/\partial z_{i}$ and 〈f〉=∫fpdx, moreover summation over repeated indices is understood)

(7) $$l_{\alpha }=h_{\alpha }-\partial_{\alpha }\psi =h_{\alpha }-\langle h_{\alpha }\rangle ,\;\alpha =1,\ldots a.$$

The last equality $\partial_{\alpha }\psi =\langle h_{\alpha }\rangle$ holds if we assume that the integrability condition $\langle h_{\alpha }\rangle$ is satisfied for every α. It is not restrictive to assume that $\langle h_{\alpha }\rangle =0$ therefore the regularity condition 2. holds if and only if the *d* functions $h_{\alpha }$ are linearly independent over *X*, which again follows from (2).

One can show (see [22,27]) that every smooth diffeomorphism θ↦m(θ) give an equivalent parameterization of the elements of the exponential family. In this sense E has the structure of a smooth manifold, called statistical manifold. Another coordinate system for E (we will denote it with p=p(x;η)) is provided by the so called *expectation* parameters $\eta \in E\subset R^{a}$ defined by (here ${\left(\partial_{\theta }\psi \right)}_{i}=\partial \psi /\partial \theta_{i})$

$$\eta =\partial_{\theta }\psi \left(\theta \right)=\int_{X}h\left(x\right)p\left(x;\theta \right)dx.$$

Since ψ is a convex function, the gradient map $\theta \mapsto \partial_{\theta }\psi \left(\theta \right)$ is globally invertible with inverse $\theta =\hat{\theta }\left(\eta \right)$ which is also a gradient map $\hat{\theta }\left(\eta \right)=\partial_{\eta }\varphi \left(\eta \right)$, where

(8) $$\varphi \left(\eta \right)=\hat{\theta }\left(\eta \right)\cdot \eta -\psi \left(\hat{\theta }\left(\eta \right)\right)$$

is the Legendre transform of ψ (see [22]).

## 3. Exponential Families Depending on External Control Parameters

These statistical models are introduced by supposing that the observables *h* that defines an exponential family depend on so-called *external* parameters $u\in U\subset R^{b}$, which are to be distinguished from the natural parameters θ. These generalized exponential families arise naturally when one applies the Maximum Entropy formalism to equilibrium Statistical Mechanics, that we briefly recall here (see E.T. Jaynes books [1,28]).

It is well known that when the information consist of the average values of some random variables $h_{\alpha }$ describing observables of interest for the system, the maximum entropy probability densities are exponential families. Indeed, if we introduce the Shannon entropy functional for a probability density p∈P(X)

(9) $$H\left(p\right)=-\int_{X}p\ln pdx,$$

then the probability density that maximize *H* on the set of probability densities that satisfy the constraints $\langle h\rangle =\int_{X}hpdx=c\in R^{a}$ has the form of an exponential family of the form in (4) with k=0. If the observables of interest for the system h=h(x,u) depend on extra parameters, the exponential family inherits naturally a dependence on the external parameters, see (15) below. Typical examples of external parameters are the magnetic or electric field applied to the system or the length of a polymer chain (see [12,29]). Also, for a quantum system confined in an infinite square well potential, the discrete energy levels $h_{i}$ depends on the width *L* of the well. Another typical example of a thermodynamic system subject to an external parameter is an ideal gas in a container of variable volume *V*; however in this case the parameter *V* affects the state space X=X(V) and not the observables *h* therefore this important system it is not described by a generalized exponential family (see [21] for a discussion of this point).

An important difference between the natural parameters θ and the external ones *u* is that the former are the Lagrange multipliers associated to the constraints when one solves the constrained extremization problem for *H* using Lagrange multipliers method, while the latter are parameters in the problem formulation that can be controlled by an agent external to the system under consideration. This difference is displayed when we consider the variation of 〈h〉 for p=p(x;θ,u). If we suppose, as we will always do, that we can exchange the order of integration and differentiation with respect to a parameter, we have

(10) $$d\langle h\rangle =\left(\int_{X}h\partial_{\theta }pdx\right)d\theta +\left(\int_{X}p\partial_{u}hdx\right)du=dQ+\langle \partial_{u}h\rangle$$

where dQ has the meaning of generalized heat exchanged and $\langle \partial_{u}h\rangle$ of generalized work exchanged (see [28]). Moreover, while the value of the external parameters *u* is controlled and can be varied by an agent external to the system, the value of the natural parameters θ can be varied only by putting the system in contact with an heath bath at a prescribed value of the inverse temperature θ (see again [28]).

The Kullback-Leibler divergence, also called relative entropy (see [27]) is defined for p,q∈P(X) and q>0 as

$$D\left(p|q\right)=\int_{X}p\left(x\right)\log\frac{p(x)}{q(x)}dx.$$

It is well known that the probability density $\hat{p}$ that minimize *D* on the set of probability densities that satisfy the constraints $\langle h\rangle =\int_{X}hpdx=c\in R^{a}$ has the form of an exponential family as in (4) with $q=e^{K}>0$

(11) $$\hat{p}\left(x;\theta \right)=e^{\theta \cdot h(x)-\psi (\theta )+k(x)}.$$

The probability distribution $\hat{p}$ is the distribution that gives the minimum information gain when one wants to update the current statistical description of the system given by *q* using the new available information 〈h〉=c. We will refer in the sequel to this as the minimum Relative Entropy principle. The parameters θ of $\hat{p}\left(x;\theta \right)$ in (4) are uniquely determined as $\theta =\hat{\theta }\left(c\right)$ by the constraint conditions

(12) $$\langle h\rangle =\partial_{\theta }\psi \left(\theta \right)=c$$

since the gradient map $\theta \mapsto \partial_{\theta }\psi$ is invertible. Note that for θ=0 we have p(x;0)=q(x) therefore the case $\hat{\theta }\left(c\right)=0$ corresponds uniquely to the constraint value $c=\int_{X}hqdx$ meaning that the constraints do not represent a new piece of information on the system. We will use this fact in the following.

Having exposed the motivations for considering these probability distributions, in the sequel we will investigate the geometrical properties of exponential families with external parameters or controlled exponential families for short.

### 3.1. Exponential Families with External Parameters

Let $U\subset R^{b}$ be the external parameter space and consider the *a* observables

$$h_{\alpha }:X\times U⟶R.$$

Let k(x) be a function on *X* and define the free energy $\psi :Z\subset R^{d}$, ψ=ψ(θ,u), d=a+b

(13) $$\psi \left(\theta ,u\right)=\ln\int_{X}e^{\theta \cdot h(x,u)+k(x)}dx$$

where the parameter space *Z* is the subset of $R^{d}$ where $e^{\psi }<+\infty$. We suppose that

(i) *Z* is open and we introduce the map
  (14) π:Z⟶U,π(θ,u)=u.
  We consider the following subset of the infinite dimensional space P(X)
  (15) $$F=\{\;p\left(x;\theta ,u\right)=e^{\theta \cdot h(x,u)-\psi (\theta ,u)+k(x)},\;\left(\theta ,u\right)\in Z\}\subset P\left(X\right)$$
  and we suppose that
(ii) for every fixed u∈π(Z) the set E(u)⊂F
  (16) $$E\left(u\right)=\{\;p\left(x;\theta \right)=e^{\theta \cdot h(x,u)-\psi (\theta ,u)+k(x)},\;\left(\theta ,u\right)\in \pi^{-1}\left(u\right)\}\subset P\left(X\right)$$
  is an exponential family. As a consequence $\pi^{-1}\left(u\right)$ is a convex subset in θ and $h_{\alpha }\left(x,u\right),1$ are a+1 functions linearly independent over *X*.

A natural question is to ask if the set F can be seen as a foliated manifold whose leaves are the statistical manifolds E(u). Note however that if θ=0 is allowed (that is $\int_{X}e^{k}dx<+\infty )$ we have for θ=0 in (13) ψ(0)=ψ(0,u) and $p\left(x;0,u\right)=e^{k(x)-\psi (0)}$ for every u∈π(Z) therefore

$$e^{k(x)-\psi (0)}\in E\left(u\right)\cap E\left(u^{\prime }\right)\;\forall \;u,u^{\prime }\in \pi \left(Z\right).$$

So the statistical manifold leaves are not disjoint.

A second natural question to ask is if F can be given the structure of a regular statistical model. To this we need to check conditions 1. and 2. in the Definition 1 above. Concerning injectivity condition 1. for the map z↦f(z) we have that $f\left(0,u\right)=e^{k-\psi (0)}$ for all u∈U so injectivity condition 1. may fail for controlled exponential families at θ=0. However, if we recall the statistical mechanics interpretations of controlled exponential families made in Section 3 and in particular in (12), we can consider the point of singularity θ=0 outside the domain of application of the statistical model (see however [20] for a discussion of this point). If we assume θ≠0, due to the possibly nonlinear dependence of h(x,u) on *u*, condition (6) to assess injectivity for a controlled exponential family becomes

(17) $$p_{z}=\theta \cdot h\left(x,u\right)-\psi \left(\theta ,u\right)=\theta^{\prime }\cdot h\left(x,u^{\prime }\right)-\psi \left(\theta^{\prime },u^{\prime }\right)=p_{z^{\prime }}\;\forall x\in X\;\;\Rightarrow \;\;\theta =\theta^{\prime },u=u^{\prime }.$$

Condition (17) seems hard to satisfy even if we assume hypothesis (ii) as the following example shows. Suppose that the observables *h* depends *linearly* on *u*

(18) $$h_{\alpha }\left(x,u\right)=A_{\alpha k}\left(x\right)u_{k}$$

and (see (ii)) suppose that the d+1 functions in (18) $h_{\alpha }\left(x,u\right),1$ are linearly independent over *X* for every fixed *u*. Note that the elements of F in (15) depend on θ, *u* through the scalar quantity θ·Au. To prove injectivity of the map $z\mapsto p_{z}$, z=(θ,u) we need to prove that if $z\neq z^{\prime }$

$$\theta \cdot A\left(x\right)u\;\neq \;\theta^{\prime }\cdot A\left(x\right)u^{\prime }$$

as functions on *X*. But this is not true if for example $\theta^{\prime }=\lambda \theta$ and $u^{\prime }=u/\lambda$ for λ≠0. So the model (18) is singular. This should not be a surprise because elements of the family F are not characterized by the observables $A_{\alpha k}\left(x\right)$ but by the linear space spanned by the $A_{\alpha k}\left(x\right)$. Indeed, if we set $\theta =B\theta^{\prime }$ and $u=Cu^{\prime }$ where *B* and *C* are nonsingular square matrices, then

$$\theta \cdot Au=\theta^{T}Au={\left(B\theta^{\prime }\right)}^{T}ACu^{\prime }=\theta^{\prime T}B^{T}ACu^{\prime }=\theta^{\prime T}A^{\prime }u^{\prime }=\theta^{\prime }\cdot A^{\prime }u^{\prime }$$

hence the family F is equally described by $A^{\prime }=B^{T}AC$ with respect to the parameters $\left(\theta^{\prime },u^{\prime }\right)$. Another lesson we can draw from this example is that for an exponential family linearly dependent in the external parameters, the distinction between natural and external ones is lost, as their role can be interchanged.

All that said, we stipulate that

**Definition** **2.** *F in (15) is an exponential family depending on the parameters z=(θ,u) if (i) for every fixed u the set E(u) is an exponential family and (ii) F is a regular d=a+b statistical model for a suitable choice of the open parameter set $Z\subset R^{a}\times U$.*

In the case of an exponential family (15) depending on natural and external parameters in addition to *a* natural parameters score base vectors

(19) $$l_{\alpha }=\frac{\partial \ln p}{\partial \theta_{\alpha }}=h_{\alpha }-\partial_{\alpha }\psi =h_{\alpha }-\langle h_{\alpha }\rangle$$

we have *b* external parameters score base vectors

(20) $$l_{k}=\frac{\partial \ln p}{\partial u_{k}}=\theta_{\alpha }\partial_{k}h_{\alpha }-\partial_{k}\psi =\theta_{\alpha }\left(\partial_{k}h_{\alpha }-\langle \partial_{k}h_{\alpha }\rangle \right)=\theta_{\alpha }L_{\alpha k}.$$

Note that $\langle l_{\alpha }\rangle =0$ and $\langle l_{k}\rangle =0$ because $\langle L_{\alpha k}\rangle =0$. Moreover, one can always assume that $\langle h_{\alpha }\rangle =0$ and $\langle \partial_{k}h_{\alpha }\rangle =0$ therefore the regularity condition 2. above holds if and only if the a+b functions

(21) $$h_{\alpha }\left(x,u\right),\;\theta_{\alpha }\frac{\partial h_{\alpha }}{\partial u_{k}}\left(x,u\right)$$

are linearly independent over *X*.

### 3.2. Fisher Metric for an Exponential Family with External Parameters

Regular statistical models can be endowed with a Riemannian metric defined on their parameter space *Z*. This is called Fisher metric [30] and it has the form

(22) $$g_{ij}\left(z\right)=\langle l_{i}l_{j}\rangle =\int_{X}\frac{\partial l}{\partial z_{i}}\frac{\partial l}{\partial z_{j}}pdx.$$

The Fisher matrix is symmetric and positive definite therefore it defines a Riemannian metric on Z (see [24], p. 24). In fact we have

(23) $$g_{ij}v_{i}v_{j}=\langle l_{i}l_{j}v_{i}v_{j}\rangle =\langle {\left(l_{i}v_{i}\right)}^{2}\rangle =0\;\Leftrightarrow \;l_{i}v_{i}=0\;\Leftrightarrow \;v_{i}=0\;\forall \;i$$

since the score vectors $l_{i}$ are linearly independent over *X*. Note also (see [24]) that *g* is invariant with respect to change of coordinates in the state space *X* and covariant (as an order 2 tensor) with respect to change of coordinates in the parameter space Z.

The elements of the Fisher matrix (22) relative to an exponential family with external parameters (15) can be detailed as follows: using (19)

(24) $$g_{\alpha \beta }=\langle l_{\alpha }l_{\beta }\rangle =\langle \left(\langle h_{\alpha }\rangle -h_{\alpha }\right)\left(\langle h_{\beta }\rangle -h_{\beta }\right)\rangle =\mathrm{cov}\left(h_{\alpha },h_{\beta }\right);$$

we also have from (20)

(25) $$g_{\alpha k}=\langle l_{\alpha }l_{k}\rangle =\langle \left(\langle h_{\alpha }\rangle -h_{\alpha }\right)\theta_{\beta }\left(\langle \partial_{k}h_{\beta }\rangle -\partial_{k}h_{\beta }\right)\rangle =\theta_{\beta }\mathrm{cov}\left(h_{\alpha },\partial_{k}h_{\beta }\right)$$

and

(26) $$g_{km}=\langle l_{k}l_{m}\rangle =\langle \theta_{\alpha }\left(\langle \partial_{k}h_{\alpha }\rangle -\partial_{k}h_{\alpha }\right)\theta_{\beta }\left(\langle \partial_{m}h_{\beta }\rangle -\partial_{m}h_{\beta }\right)\rangle =\theta_{\alpha }\theta_{\beta }\mathrm{cov}\left(\partial_{k}h_{\alpha },\partial_{m}h_{\beta }\right).$$

It is useful to set

$$A_{\alpha \beta }=g_{\alpha \beta },\;M_{\alpha k}=g_{\alpha k},\;B_{km}=g_{km}$$

and introduce a block representation of the symmetric (a+b)-dimensional Fisher matrix *g* as

(27) $$g\left(z\right)=\left(\begin{array}{cc} A & M\\ M^{T} & B \end{array}\right).$$

We now give the expression of the Fisher metric coefficients using the free entropy function ψ in (13), which is also called the moment generating function because its derivative with respect to the θ parameters give the different moments of the random variables *h*. We thus have the well know relation

(28) $$\partial_{\beta }\partial_{\alpha }\psi =\mathrm{cov}\left(h_{\alpha },h_{\beta }\right)=g_{\alpha \beta }.$$

By direct computation on (13) we have also

$$\partial_{k}\partial_{\alpha }\psi =\theta_{\beta }\mathrm{cov}\left(h_{\alpha },\partial_{k}h_{\beta }\right)+\langle \partial_{k}h_{\alpha }\rangle$$

hence

(29) $$g_{\alpha k}=\partial_{k}\partial_{\alpha }\psi -\langle \partial_{k}h_{\alpha }\rangle .$$

Moreover we have

(30) $$\partial_{k}\partial_{m}\psi =\theta_{\alpha }\theta_{\beta }\mathrm{cov}\left(\partial_{k}h_{\alpha },\partial_{m}h_{\beta }\right)+\theta_{\alpha }\langle \partial_{k}\partial_{m}h_{\alpha }\rangle$$

hence

(31) $$g_{km}=\partial_{k}\partial_{m}\psi -\theta_{\alpha }\langle \partial_{k}\partial_{m}h_{\alpha }\rangle .$$

We see that, unlike the case of natural parameters θ, second order derivatives of the free entropy ψ with respect to mixed or external parameters do not coincides with the elements of Fisher matrix.

**Example** **1.** *As a toy model, we introduce the following example of a controlled exponential family. Let X=[0,+∞) and U=[0,+∞) and consider the two observables where x∈X,u∈U*

(32) $$h_{1}\left(x\right)=\ln x,\;h_{2}\left(x,u\right)=\ln\left(x+u\right).$$

*For this example we set k(x)=−lnx. We check that we have an integrable free energy function*

$$\begin{array}{ccc} e^{\psi } & = & \int_{X}e^{\theta \cdot h+k}dx=\int_{0}^{+\infty }e^{\left(\theta_{1}-1\right)\ln x+\theta_{2}\ln\left(x+u\right)}dx\\ & = & \int_{0}^{+\infty }x^{\theta_{1}-1}{\left(x+u\right)}^{\theta_{2}}dx=u^{\theta_{1}+\theta_{2}}\Gamma \left(\theta_{1}\right)\frac{\Gamma (-\theta_{2}-\theta_{1})}{\Gamma (-\theta_{2})} \end{array}$$

*which is finite if $\theta_{1}>0$, u>0 and $\theta_{2}+\theta_{1}<0$. Here Γ(z) is the Gamma function defined as*

$$\Gamma \left(z\right)=\int_{0}^{+\infty }t^{z-1}e^{-z}dt.$$

*Note that since $e^{k}\left(x\right)=1/x$ is non integrable over X, $\theta_{1}=\theta_{2}=0$ is a non feasible value. By inspection $h_{1},h_{2},1$ are linearly independent over X for every fixed u, the map*

$$\left(\theta_{1},\theta_{2},u\right)\mapsto \theta_{1}\ln\left(x\right)+\theta_{2}\ln\left(x+u\right)-\ln\left(x\right)$$

*is injective. From the likelihood*

$$l=\theta_{1}\ln\left(x\right)+\theta_{2}\ln\left(x+u\right)-\ln\left(x\right)-\psi \left(\theta ,u\right),$$

*the elements of the score base are*

$$l_{1}=\ln\left(x\right)-\partial_{1}\psi ,\;l_{2}=\ln\left(x+u\right)-\partial_{2}\psi ,\;l_{u}=\theta_{2}\left(\frac{1}{x+u}-\partial_{u}\psi \right)$$

*which are linearly independent over X. So the statistical model defined by (32) is a 2+1 dimensional controlled exponential family. Note that the probability density*

$$p\left(x;\theta_{1},\theta_{2},u\right)=e^{\theta_{1}h_{1}+\theta_{2}h_{2}-\psi +k}=\frac{x^{\theta_{1}-1}{\left(x+u\right)}^{\theta_{2}}}{Z(\theta_{1},\theta_{2},u)}$$

*is known as a (possible formulation of a) compound Gamma distribution; moreover, for u=1, this is the Beta distribution of second kind [31].*

*We now compute the Fisher matrix elements for this example. Let us introduce the Polygamma function $\Phi_{m}$ for m∈N*

$$\Phi_{m}\left(z\right)=\frac{d^{m}}{dz^{m}}\Phi_{0}\left(z\right),\;\Phi_{0}\left(z\right)=\frac{d}{dz}\ln\Gamma \left(z\right)=\frac{\Gamma^{\prime }\left(z\right)}{\Gamma (z)}.$$

*We have*

$$\psi \left(\theta_{1},\theta_{2},u\right)=\left(\theta_{1}+\theta_{2}\right)\ln u+\ln\frac{\Gamma \left(\theta -1\right)\Gamma \left(-\theta_{1}-\theta_{2}\right)}{\Gamma (-\theta_{2})}$$

*and from relation (28) above we have*

$$\begin{array}{ccc} g_{11} & = & \partial_{1}\partial_{1}\psi =\Phi_{1}\left(\theta_{1}\right)+\Phi_{1}\left(-\theta_{1}-\theta_{2}\right)\\ g_{12} & = & \partial_{1}\partial_{2}\psi =\Phi_{1}\left(-\theta_{1}-\theta_{2}\right)\\ g_{22} & = & \partial_{1}\partial_{1}\psi =-\Phi_{1}\left(-\theta_{2}\right)+\Phi_{1}\left(-\theta_{1}-\theta_{2}\right) \end{array}$$

*so the A block of g depends only on θ. Moreover from (29) and (31) we have*

$$\begin{array}{ccc} g_{1u} & = & \partial_{u}\partial_{1}\psi -\langle \partial_{u}h_{1}\rangle =\partial_{u}\partial_{1}\psi =\frac{1}{u}\\ g_{2u} & = & \partial_{u}\partial_{2}\psi -\langle \partial_{u}h_{2}\rangle =\frac{1}{u}+\frac{\theta_{1}+\theta_{2}}{\theta_{2}u}=-\frac{\theta_{1}}{u\theta_{2}}\\ g_{uu} & = & \partial_{u}\partial_{u}\psi -\theta_{\alpha }\langle \partial_{u}\partial_{u}h_{\alpha }\rangle =\frac{\left(\theta_{1}+\theta_{2}\right)\theta_{1}}{u^{2}\left(\theta_{2}-1\right)}. \end{array}$$

## 4. A Synopsis of Ehresmann Connections

On a smooth fibration π:M⟶N, where *M*,N are smooth manifolds, with dimM=m, dimN=n, the set VM=kerTπ of the vectors that project onto the null space of TN is an integrable subbundle of TM called the vertical bundle.

An Ehresmann connection (see e.g., [32]) on π:M⟶N is the assignment of a distribution HM transversal to VM, so that HM⊕VM=TM. The elements of HM are the horizontal vectors; since Tπ restricted to HM is an isomorphism, it has a fiberwise defined inverse, the horizontal lift: $\mathrm{hor}:T_{\pi (z)}N⟶T_{z}M$,$\;\mathrm{hor}\left(X\right)\in H_{z}M$. Let $X=X^{h}+X^{v}$ be the splitting of a vector in $T_{z}M$ into its horizontal and vertical component. The projection on VM with respect to the horizontal subspace defines the vector–valued connection one-form

(33) $$\omega :TM⟶VM,\;\omega \left(z\right)\left(X\right)=X^{v},$$

whose kernel is the horizontal distribution. The assignment of an horizontal distribution, of an horizontal lift operator or of a connection one-form are equivalent ways to define a connection on π:M⟶N. The *curvature* of the connection is the VM–valued two-form defined as

(34) $$\Omega \left(X,Y\right)=-\omega (\left[X^{h},Y^{h}\right])$$

which shows that the curvature measures the failure of the horizontal distribution to be integrable. Moreover, the curvature relates the Lie brackets of vector fields X,Y on the base manifold *N* with the Lie bracket of their horizontal lifts through the formula

(35) $$\Omega \left(\mathrm{hor}X,\mathrm{hor}Y\right)={\left[\mathrm{hor}X,\mathrm{hor}Y\right]}_{M}-\mathrm{hor}{\left[X,Y\right]}_{N}.$$

Again, we find that if the curvature is vanishing the horizontal distribution, spanned by vectors of the type horX, is *involutive* hence integrable. Next we give the local expressions of a connection in a fibered chart. Let z=(x,y) be a fibered chart on U⊂M, π(x,y)=y. Then the vertical space is, α=1,…,a=dimM−dimN,

(36) $$V_{z}U=\ker\;T_{z}\pi =\mathrm{span}\left\{\frac{\partial }{\partial x^{\alpha }}\right\}$$

and the connection one-form ω is:

(37) $$\omega =\omega^{\alpha }⊗\frac{\partial }{\partial x^{\alpha }},\;\omega^{\alpha }=dx^{\alpha }+A_{l}^{\alpha }\left(z\right)dy^{l}.$$

The $A_{l}^{\alpha }\left(z\right)$ are the connection’s coefficients. The horizontal vectors have the coordinate expression

$$X\in HM\;\Leftrightarrow \;\omega \left(X\right)=0,\;\Leftrightarrow \;X^{l}=X^{l}\left(\frac{\partial }{\partial y^{l}}-A_{l}^{\alpha }\frac{\partial }{\partial x^{\alpha }}\right)$$

while the horizontal lift of a base vector $U=U^{l}\frac{\partial }{\partial y^{l}}\in T_{\pi (z)}N$ has the form

(38) $${\left(\mathrm{hor}\;U\right)}^{l}\left(z\right)=U^{l}\left(\frac{\partial }{\partial y^{l}}-A_{l}^{\alpha }\frac{\partial }{\partial x^{\alpha }}\right).$$

We now specialize the above relations to the important case where the horizontal distribution $H_{z}M$ is defined to be the *g*-orthogonal of $V_{z}M$ with respect to a Riemannian metric *g* on *M*. Referring to a block representation of the metric *g* in the coordinates (x,y) like the above one (27) for (θ,u) we ask that every $X^{h}\in H_{z}M$, $\;X^{h}=\left(-A_{l}^{\alpha }U^{l},U^{l}\right)$ be orthogonal to all $X^{v}\in V_{z}M$, $X^{v}=\left(W,0\right)$. As a consequence

(39) $$g\left(X^{v},X^{h}\right)=W\cdot \left(-AAU+MU\right)=0\;\forall \;W\;\Leftrightarrow \;A=A^{-1}M.$$

The connection one-form (37) becomes from (39)

(40) $$\omega^{\alpha }=dx^{\alpha }+A_{l}^{\alpha }\left(z\right)dy^{l},\;\mathrm{where}\;A_{l}^{\alpha }={\left(A^{-1}M\right)}_{l}^{\alpha }$$

and it is called mechanical connection in the control theory for mechanical systems, where *g* is the kinetic energy of a mechanical system. In the orthogonal splitting case the metric *g* has the simpler form by (39)

$$g\left(X,Y\right)=g\left(X^{v}+X^{h},Y^{v}+Y^{h}\right)=g\left(X^{v},Y^{v}\right)+g\left(X^{h},Y^{h}\right)$$

Since $X^{v}=\left(\omega \left(X\right),0\right)$ and using again the block representation (27) of *g* we have

$$g\left(X^{v},Y^{v}\right)=A_{\alpha \beta }\left(z\right)\omega^{\alpha }\left(X\right)\omega^{\beta }\left(Y\right)$$

and

$$g\left(X^{h},Y^{h}\right)={\left(-AU,U\right)}^{T}g\left(-AV,V\right)=U^{T}KV$$

where $K=B-M^{T}A^{-1}M=K^{T}$ hence

(41) g(z)dz⊗dz=A(z)ω(·)⊗ω(·)+K(z)dy⊗dy.

and

(42) $$g=\left(\begin{array}{cc} A & 0\\ 0 & K \end{array}\right).$$

### Parallel Transport Equation

Let γ:[0,T]→N be a smooth path in the base manifold and let $z_{0}\in \pi^{-1}\left(\gamma \left(0\right)\right)$. The parallel transport equation is the following ODE for the horizontal lift vector field

(43) $$\dot{z}=\frac{dz}{dt}=\mathrm{hor}\left(\dot{\gamma }\right),\;z\left(0\right)=z_{0}$$

with local expression

(44) $${\dot{x}}_{\alpha }=-A_{\alpha }^{l}\left(x,\gamma \right){\dot{\gamma }}_{l},\;{\dot{y}}_{l}={\dot{\gamma }}_{l}.$$

The connection is called complete if the parallel transport equation has a solution defined on the whole [0,T]. If in (41) we have K=K(y) then the metric *g* is called bundle-like metric. The main geometric consequence is that if we introduce the Riemannian manifold (N,K) then the horizontal lift is an isometry and the solution z(t) of the parallel transport equation is a curve that projects over γ of the same length.

## 5. Some Applications of Exponential Families with External Parameters

In this Section we apply the geometric framework of the previous Section 4 to the fibration π:Z⟶U, π(z,u)=u introduced in (14). We can also consider the inverse φ of the map $z\mapsto f\left(z\right)=p_{z}$ and introduce the fibration

$$F\overset{\varphi }{⟶}Z\overset{\pi }{⟶}U,\;\tilde{\pi }=\pi \circ \varphi .$$

Since ${\tilde{\pi }}^{-1}\left(u\right)=E\left(u\right)$, fibers of $\tilde{\pi }$ are exponential families for every fixed value of the external parameters. One can show that the orthogonal splitting of TZ induces and orthogonal splitting of TF with respect to the Fisher metric (see [21]).

### 5.1. Thermodynamic Length

Let t↦z(t)=(θ(t),u(t))∈Z, t∈[0,T] be a path in parameter space. Define the time-dependent relative entropy along the path as D(t)=D(p(z(t))|p(z(0)) and compute the Taylor expansion of D(t) at t=0. A direct computation shows that D(0)=0, $D^{\prime }\left(0\right)=0$ hence

$$D\left(dt\right)=D\left(0\right)+D^{\prime }\left(0\right)dt+\frac{1}{2}D^{\prime \prime }\left(0\right)dt^{2}+O\left(dt^{3}\right)=\frac{1}{2}D^{\prime \prime }\left(0\right)dt^{2}+O\left(dt^{3}\right)=\frac{1}{2}{∥\dot{z}∥}_{g}^{2}+O\left(dt^{3}\right)$$

where ${∥\dot{z}∥}_{g}^{2}$ is the scalar product with respect to the Fisher metric in (Z,g). It holds that

$$\begin{array}{ccc} {∥\dot{z}∥}_{g}^{2} & = & \dot{z}\cdot g\left(z\right)\dot{z}\langle l_{i}l_{j}\rangle {\dot{z}}_{i}{\dot{z}}_{j}=\langle l_{i}l_{j}{\dot{z}}_{i}{\dot{z}}_{j}\rangle \\ & = & \int_{X}p\partial_{i}\ln p\;\partial_{j}\ln p\;{\dot{z}}_{i}{\dot{z}}_{j}dx=\int_{X}p\left(\frac{1}{p}\partial_{i}p\frac{1}{p}\partial_{j}p\right){\dot{z}}_{i}{\dot{z}}_{j}dx\\ & = & \int_{X}\frac{1}{p}{\left(\frac{dp}{dt}\left(z\left(t\right)\right)\right)}^{2}dx. \end{array}$$

The quantity $2D\left(dt\right)={∥\dot{z}∥}_{g}^{2}$ can be related to the entropy change rate dσ/dt of the heat bath and to the total system entropy production rate dF/dt in a non quasi-static evolution of the system by the formula (see [14])

$${∥\dot{z}∥}_{g}^{2}=\langle \frac{d\sigma }{dt}\rangle -\langle \frac{dF}{dt}\rangle \geq 0.$$

Therefore ${∥\dot{z}∥}_{g}^{2}$ is a measure of the system entropy production rate $d\sigma_{sys}/dt$ in a non-quasi static evolution of the system. When integrated along the finite time evolution protocol z(t), the quantity

$$2C=\int_{0}^{T}{∥\dot{z}∥}_{g}^{2}dt=\int_{0}^{T}\left[\langle \frac{d\sigma }{dt}\rangle -\langle \frac{dF}{dt}\rangle \right]dt$$

is called action of the path and can be interpreted as the thermodynamic cost (loss in the entropy transfer due to the system entropy production) associated to the protocol therefore it is a measure of the dissipated (non available) work. The quantity (see [11,12,15])

$$L\left(z\right)=\int_{0}^{T}{∥\dot{z}∥}_{g}\left(t\right)dt$$

is called the thermodynamic length of the path z(·). By the Cauchy-Schwartz inequality one obtains the inequality (see [14])

$$T2C\geq {\left(L\left(z\right)\right)}^{2}$$

showing that the thermodynamic length (TL) gives a lower bound on the dissipated work in a non quasi-static evolution of the system [11,15]. The above relation is used when studying the controlled evolution of classical and quantum small thermodynamic systems, e.g., molecular motors (see [15]).

Using the representation (41) of the scalar product with respect to the Fisher metric *g* we have the interesting formula for the TL of a controlled exponential family

$$L\left(z\right)=\int_{0}^{T}{∥\dot{z}∥}_{g}\left(t\right)dt=\int_{0}^{T}{\left(A_{\alpha \beta }\left(z\right)\omega^{\alpha }\left(\dot{z}\right)\omega^{\beta }\left(\dot{z}\right)+K_{lm}\left(z\right){\dot{u}}^{l}{\dot{u}}^{m}\right)}^{\frac{1}{2}}dt.$$

In particular, if the path *z* is the horizontal lift of a path *u* in the external parameter space then $\dot{z}=\mathrm{hor}\left(\dot{u}\right)$ and $\omega (\dot{z})=0$. If moreover the metric *g* is bundle-like with respect to the fibration π we have K(z)=K(π(z)) and the thermodynamic length can be expressed as

$$L\left(z\right)=L\left(u\right)=\int_{0}^{T}\sqrt{K\left(u\right)\dot{u}\cdot \dot{u}}\;dt$$

showing that TL depends solely on the external parameters evolution u=u(t).

### 5.2. Isentropic Evolution Driven by External Parameters

We have recalled in Section 3 that the elements of a controlled exponential family F where $q=e^{K}\in P$ are the solution of the constrained minimization problem for the relative entropy D(p|q) of the form (11)

$$\hat{p}\left(x;c,u\right)=e^{\hat{\theta }\cdot h\left(x,u\right)-\psi \left(\hat{\theta },u\right)+k\left(x\right)}$$

where $\hat{\theta }=\hat{\theta }\left(c,u\right)$ is uniquely determined by inverting the gradient map $\partial_{\theta }\psi \left(\theta ,u\right)=c$. We have that

$$D\left(c,u\right)=D\left(\hat{p}|q\right)=\int_{X}\hat{p}\ln\frac{\hat{p}}{q}dx=\hat{\theta }\cdot \langle h\rangle -\psi =\hat{\theta }\cdot \partial_{\theta }\psi -\psi =-S\left(c,u\right)$$

where $S\left(c,u\right)=\psi -\theta \cdot \partial_{\theta }\psi$ is the entropy of the statistical system when the information on the system is described by the constraint 〈h〉=c. In the following we consider D(c,u) as a function of (θ,u) knowing that θ is in a one-to-one correspondence with *c*.

Let us compute the differential of D(θ,u) corresponding to a infinitesimal variation of the parameters z=(θ,u). We have

(45) $$dD\left(\theta ,u\right)=D\left(\hat{p}|q\right)=\partial_{\theta }Dd\theta +\partial_{u}Ddu$$

More in detail, using (28) we obtain

$$\partial_{\beta }D=\partial_{\beta }\left(\theta_{\alpha }\partial_{\alpha }\psi -\psi \right)=\partial_{\beta }\psi +\theta_{\alpha }\partial_{\beta }\partial_{\alpha }\psi -\partial_{\beta }\psi =\theta_{\alpha }g_{\alpha \beta }$$

and using (29) we obtain

$$\partial_{k}D=\partial_{k}\left(\theta_{\alpha }\partial_{\alpha }\psi -\psi \right)=\theta_{\alpha }\partial_{k}\partial_{\alpha }\psi -\partial_{k}\psi =\theta_{\alpha }(g_{\alpha k}+\langle \partial_{k}h_{\alpha }\rangle -\theta_{\alpha }\langle \partial_{k}h_{\alpha }\rangle =\theta_{\alpha }g_{\alpha k}$$

so collecting the results and using (40) we have

$$dD\left(z\right)=\theta_{\alpha }\left(g_{\alpha \beta }d\theta^{\beta }+g_{\alpha k}du_{k}\right)=\theta_{\alpha }\left(A_{\alpha \beta }d\theta_{\beta }+M_{\alpha k}du_{k}\right)=\theta_{\alpha }A_{\alpha \beta }\omega^{\beta }$$

and the following proposition holds

**Proposition** **1.** *(1) The variation of entropy for an infinitesimal change in the parameters z=(θ,u) can be expressed using the g-orthogonal Ehresmann connection ω on π:Z⟶U*

(46) $$-dS=dD=\theta_{\alpha }A_{\alpha \beta }\omega^{\beta }=\theta_{\alpha }\partial_{\alpha }\partial_{\beta }\psi \omega^{\beta }$$

*(2) the change in entropy for the system along a given path z=z(t), t∈[0,T] in parameter space is given by*

$$\Delta S\left(z\right)=\int_{0}^{T}dS\left(\dot{z}\right)dt=-\int_{0}^{T}\theta_{\alpha }\left(t\right)A_{\alpha \beta }\left(z\left(t\right)\right)\omega^{\beta }\left(\dot{z}\right)dt$$

*(3) since $\omega (\dot{z})=0$ for an horizontal path, the horizontal lift $\dot{z}=\mathrm{hor}\dot{\gamma }$ of a path γ in the external parameter space U gives an isentropic (ΔS=0) evolution of the system.*

Note that the horizontal lift do not represent all the possible isentropic evolution of the system. These are characterized by the weaker (with respect to $\omega (\dot{z})=0$) condition $\theta \cdot A\omega (\dot{z})=0$. Let us investigate this condition using the general relation (10) that we can now write as

$$d\langle h_{\alpha }\rangle =d\left(\partial_{\alpha }\psi \right)=\partial_{\alpha }\partial_{\beta }\psi d\theta_{\beta }+\partial_{k}\partial_{\alpha }\psi du_{k}=A_{\alpha \beta }\omega^{\beta }+\langle \partial_{k}h_{\alpha }\rangle du_{k}=dQ_{\alpha }+dW_{\alpha }.$$

If we want to gain insight into the above relation using a thermodynamic analogy, then $h_{\alpha }$ is the α-type energy, $dQ_{\alpha }=A_{\alpha \beta }\omega^{\beta }$ is the α-type heat exchanged and $dW_{\alpha }$ the α-type work exchanged. If we interpret the natural parameters θ as the α-type inverse temperature $\theta_{\alpha }=1/T_{\alpha }$ then (46) display as

$$-dS=\theta_{\alpha }A_{\alpha \beta }\omega^{\beta }=\sum_{\alpha }\frac{dQ_{\alpha }}{T_{\alpha }}.$$

Therefore an horizontal path corresponds to the condition $dQ_{\alpha }=0$ for all α and certainly it represents an isentropic evolution of the system, but we can have an isentropic evolution even if $dQ_{\alpha }\neq 0$ if the heat fluxes divided by their temperatures have a zero sum. As a final remark, note that in the exponential family we have the scalar product θ·h hence the inverse temperature vector $\theta \in R^{a}$ should be seen as an element of the dual space of the $h\in R^{a}$ vector space and not as a point in a local coordinate chart. See [33] on this point.

## 6. Information Geometry of Gradient Systems

In this Section we consider a class of evolution problems described by a Fokker-Planck type equation (FPE) on a regular connected domain $X\subset R^{n}$ which is open and bounded. We write FPE as in [34] (i,j=1,…,n, repeated indices are summed)

(47) $$\partial_{t}p=-\partial_{i}\left(D_{i}p\right)+\partial_{i}\partial_{j}\left(D_{ij}p\right)=-\nabla \cdot S$$

where $D_{i}\left(x\right)$ is the drift field, $D_{ij}\left(x\right)$ is the symmetric diffusion matrix, ∇· denotes divergence and *S* is the probability current

$$S_{i}\left(p\right)=D_{i}p-\partial_{j}\left(D_{ij}p\right),\;i=1,\ldots ,n.$$

To ensure that a solution p(x,t) is normalized to one for all t≥0 we need to ask

$$0=\frac{d}{dt}\int_{X}pdx=\int_{X}\partial_{t}pdx=-\int_{X}\nabla \cdot Sdx=-\int_{\partial X}S\cdot \nu d\sigma$$

that is S·ν=0 on ∂X. We restrict to the case that the diffusion matrix is diagonal $D_{ij}=d_{i}\left(x\right)\delta_{ij}$ and positive definite ( $d_{i}>0$) and therefore we rewrite *S* as

(48) $$S_{i}\left(p\right)=D_{i}p-\partial_{i}\left(d_{i}p\right)=p\left[D_{i}-d_{i}\partial_{i}\ln\left(pd_{i}\right)\right].$$

Moreover we suppose that the drift field is of the form

(49) $$D_{i}\left(x\right)=d_{i}\left(x\right)\partial_{i}\phi \left(x\right)$$

where ϕ is a function defined on *X*. A stationary solution $p_{\infty }$ of FPE is obtained if we have $S_{i}\left(p_{\infty }\right)=0$ for all *i* that is from (48)

(50) $$d_{i}\left[\partial_{i}\left(\phi -\ln(p_{\infty }d_{i})\right)\right]=0.$$

One can show ([34], Chapter 6) that in this setting the stationary solution to FPE (47) is unique. We can rewrite the FPE using $p_{\infty }$ from (48) and (49) as follows

$$\begin{array}{ccc} \partial_{t}p & = & -\partial_{i}S_{i}=\partial_{i}\left[pd_{i}\partial_{i}\left(-\phi +\ln\left(pd_{i}\right)\right)\right]\\ & = & \partial_{i}\left[pd_{i}\partial_{i}\left(-\ln\left(p_{\infty }d_{i}\right)+\ln\left(pd_{i}\right)\right)\right]=\partial_{i}\left[pd_{i}\partial_{i}\left(\ln\frac{p}{p_{\infty }}\right)\right] \end{array}$$

or in compact notation as

(51) $$\partial_{t}p=\nabla \cdot \left(pD\nabla \ln\frac{p}{p_{\infty }}\;\right)$$

where *D* is the diagonal diffusion matrix. The trend to the equilibrium can be studied by computing the “distance in entropy” between a solution *p* of (47) and $p_{\infty }$. Setting $\lambda \left(p\right)=\ln\left(p/p_{\infty }\right)$ we have from (51)

$$\begin{array}{ccc} \frac{d}{dt}D\left(p|p_{\infty }\right) & = & \frac{d}{dt}\int_{X}p\ln\frac{p}{p_{\infty }}dx=\int_{X}\left(\partial_{t}p\right)\lambda \left(p\right)dx=\int_{X}\nabla \cdot \left(pD\nabla \lambda \right)\lambda dx \end{array}$$

and using the relation λ∇·X=∇·(λX)−X·∇λ where X=pD∇λ we get

(52) $$\begin{array}{ccc} \frac{d}{dt}D\left(p|p_{\infty }\right) & = & \int_{X}\left[\nabla \cdot \left(\lambda pD\nabla \lambda \right)-pD\nabla \lambda \cdot \nabla \lambda \right]dx\\ & = & \int_{\partial X}\lambda pD\nabla \lambda \cdot \nu d\sigma -\int_{X}pD\nabla \lambda \cdot \nabla \lambda dx\\ & = & -\int_{X}pD\nabla \ln\frac{p}{p_{\infty }}\cdot \nabla \ln\frac{p}{p_{\infty }}dx<0 \end{array}$$

because S·ν=pD∇λ·ν=0 on ∂X. So the distance in entropy tends to zero independently of the initial conditions. One can show that for the FPE we have (Csiszar-Kullback-Pinsker inequality, see [35], Chapter 9)

$$D\left(p|p_{\infty }\right)\geq \frac{1}{2}||p-p_{\infty }{\left|\right|}_{L_{1}}.$$

If we have a constant diffusion matrix $D_{ij}=d\delta_{ij}$, d>0, the above inequality (52) can be rewritten as

(53) $$\frac{d}{dt}D\left(p|p_{\infty }\right)=-d\int_{X}p{\left(\nabla \ln\frac{p}{p_{\infty }}\right)}^{2}dx=-d\;R\left(p|p_{\infty }\right)$$

where $R(p|p_{\infty })$ is called relative Fisher information (see [35], Chapter 9 or [36]).

A probability density $p_{\infty }$ satisfies a logarithmic Sobolev inequality (LSI) with positive constant σ>0 if

$$2\sigma D\left(p|p_{\infty }\right)\leq R\left(p|p_{\infty }\right)\;\;\forall p\in P\left(X\right).$$

If $p_{\infty }$ satisfies a Logarithmic Sobolev inequality we can prove the exponential speed of convergence to equilibrium in entropy; indeed we have $-R\left(p|p_{\infty }\right)\leq 2\sigma D\left(p|p_{\infty }\right)$ and by substitution in (53) we obtain

$$\frac{d}{dt}D\left(p|p_{\infty }\right)\leq -2d\sigma D\left(p|p_{\infty }\right)\;\Rightarrow \;D\left(p|p_{\infty }\right)\leq D\left(p_{0}|p_{\infty }\right)e^{-2d\sigma t}.$$

A sufficient condition for LSI is the following one (see [35], Chapter 9):

(LSI condition) Let *V* be a $C^{2}$ function on *X* with $\int_{X}e^{-V}dx=1$ and Hess(V)≥σI for some σ>0. Then $e^{-V}$ satisfies LSI with positive constant σ.

### 6.1. A Dynamic Approximation Problem

This section is motivated by a problem in quantitative genetics which has been dealt with in a series of papers [2,3,4]. See also Appendix A for a brief account. Here we introduce a slightly simplified version of the original model problem which has the advantage of a greater generality. Let us consider FPE (51) with a gradient drift field of the form (49) with $x=(x_{1},\ldots ,x_{n})$ and

(54) $$d_{i}\left(x\right)=d\left(x_{i}\right),\;\phi \left(x\right)=\theta \cdot h\left(x\right),\;D_{ij}=d\left(x_{i}\right)\delta_{ij},\;D_{i}\left(x\right)=d\left(x_{i}\right)\partial_{i}\left(\theta \cdot h\left(x\right)\right).$$

In this case one can prove that the stationary solution satisfying (50) of FPE (51) has the form of an exponential family of the form

(55) $$p_{\infty }\left(x\right)=p_{\theta }=e^{\theta \cdot h(x)-\psi +k(x)}\;\mathrm{where}\;k\left(x\right)=-\sum_{i}\ln d\left(x_{i}\right).$$

We are free to set the value of the natural parameters and we set $\theta =\theta_{1}$ where $\theta_{1}$ is a feasible value.

The explicit solution of FPE (51) is difficult to study and one could be content with the study of the time evolution of the average values of the observables $h_{\alpha }$ that is the functions

$$t\mapsto {\langle h_{\alpha }\rangle }_{p(t)}=\int_{X}h_{\alpha }\left(x\right)p\left(x,t\right)dx$$

along the unknown solution of FPE. With this aim, it is natural to consider the following:

(Approximation problem): to find the time evolution of the natural parameters θ=θ(t) such that the density

(56) $$p_{\theta }\left(x,t\right)=e^{\theta (t)\cdot h(x)-\psi (\theta (t))-k(x)}$$

has the same average values of the unknown solution of FPE i.e.,

(57) $${\langle h_{\alpha }\rangle }_{p(t)}={\langle h_{\alpha }\rangle }_{p_{\theta (t)}}.$$

This strategy (called Dynamic Maximum Entropy method in [3]) seems reasonable because the exponential density (55) is the maximum entropy distribution which satisfy the constraints of the form 〈h〉=c therefore it contains exactly the required amount of information needed to satisfy the average values constraint. In the following we will investigate the interplay between the following three densities: (1) the unknown solution *p* of FPE, (2) the approximating exponential density $p_{\theta }$ and (3) the exponential equilibrium density $p_{\infty }=p_{\theta_{1}}$.

#### Triangular Relation

To start with note that for (56) (dropping the explicit time dependence in θ and *p*)

(58) $$\left(b\right)\;D\left(p|p_{\theta }\right)=\int_{X}p\ln\frac{p}{p_{\theta }}dx=-H\left(p\right)-\theta \cdot {\langle h\rangle }_{p}+\psi -{\langle k\rangle }_{p}$$

therefore the condition (57) can be rewritten as

(59) $$\partial_{\theta }D\left(p|p_{\theta }\right)=-{\langle h\rangle }_{p}+\partial_{\theta }\psi \left(\theta \right)={\langle h\rangle }_{\theta }-{\langle h\rangle }_{p}=0.$$

Note that the equation $\partial_{\theta }\psi \left(\theta \right)={\langle h\rangle }_{p}$ has a unique solution θ=θ(t) for all t≥0.

Next, let us compute the distance in entropy between the solution of FPE and its stationary solution (55) with $\theta =\theta_{1}$

(60) $$\left(a\right)\;D\left(p|p_{\theta_{1}}\right)=-H\left(p\right)-\theta_{1}\cdot {\langle h\rangle }_{p}+\psi \left(\theta_{1}\right)-{\langle k\rangle }_{p}$$

and the distance between *p* and $p_{\theta }$ (here θ=θ(t) is the value of the approximating solution satisfying (59))

(61) $$\left(c\right)\;D\left(p_{\theta }|p_{\theta_{1}}\right)=\left(\theta -\theta_{1}\right)\cdot {\langle h\rangle }_{\theta }+\psi \left(\theta_{1}\right)-\psi \left(\theta \right)=\left(\theta -\theta_{1}\right)\cdot \partial_{\theta }\psi \left(\theta \right)+\psi \left(\theta_{1}\right)-\psi \left(\theta \right)$$

which coincides with the Bregman divergence (see [27]) of the convex function ψ

$$D_{\psi }\left(\theta_{1},\theta \right)=\psi \left(\theta_{1}\right)-\psi \left(\theta \right)-\left(\theta_{1}-\theta \right)\cdot \partial_{\theta }\psi \left(\theta \right)=D\left(p_{\theta }|p_{\theta_{1}}\right).$$

Collecting the above results (58) (60) (61) and summing the right hand sides we obtain the triangular relation (see [27], Theorem 1.2 or [22], Theorem 3.7)

(62) $$D\left(p|p_{\theta }\right)+D\left(p_{\theta }|p_{\theta_{1}}\right)=D\left(p|p_{\theta_{1}}\right)+\left(\theta -\theta_{1}\right)\cdot \left({\langle h\rangle }_{\theta }-{\langle h\rangle }_{p}\right).$$

It follows that

**Figure 1:**
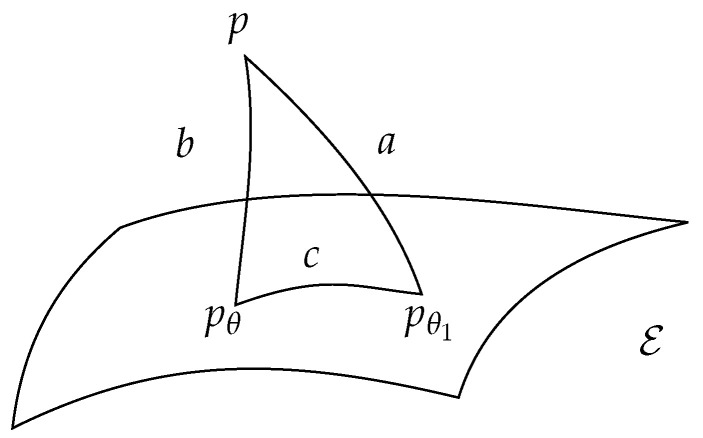
Triangular relation between $a=D(p|p_{\theta_{1}})$, $b=D(p|p_{\theta })$ and $c=D(p_{\theta }|p_{\theta_{1})}$. E is the exponential family submanifold.

**Proposition** **2.** *The function θ(t) satisfies condition (57) of the Approximation problem if and only if the following relation (called generalized Phytagorean theorem in [27]) holds (see Figure 1)*

(63) $$b+c=D\left(p|p_{\theta }\right)+D\left(p_{\theta }|p_{\theta_{1}}\right)=D\left(p|p_{\theta_{1}}\right)=a$$

*meaning that $p_{\theta (t)}$ is the* geodesic projection *of p on the exponential family (flat submanifold) E satisfying to*

$$D\left(p|p_{\theta }\right)=min\left\{D\left(p|p_{\theta^{\prime }}\right)\;:\;p_{\theta^{\prime }}\in E\right\}$$

*that is $p_{\theta (t)}$ is the best approximation of p on E with respect to the information gain.*

Note that this relation is exact and does not need the hypothesis that θ be close to $\theta_{1}$. Relation (63) characterizes θ(t) from a geometrical point of view.

We now take the time derivative of (63) with a double aim: on the one hand to find a differential relation (ODE) for θ and on the other hand to find an upper bound for the distance in entropy $b=D(p|p_{\theta })$ knowing that $a=D(p|p_{\theta_{1}})$ tends to 0, possibly with exponential speed. Note that taking the time derivative of the relation (63) b+c=a is equivalent to taking the time derivative of the relation (59), since the two are equivalent conditions on θ(t). We have from (52)

(64) $$\dot{a}=\frac{d}{dt}D\left(p|p_{\theta_{1}}\right)=-\int_{X}pD\nabla \ln\frac{p}{p_{\theta_{1}}}\cdot \nabla \ln\frac{p}{p_{\theta_{1}}}dx<0.$$

Moreover

$$\dot{b}=\frac{d}{dt}D\left(p|p_{\theta }\right)=\frac{d}{dt}\int_{X}p\ln\frac{p}{p_{\theta }}dx=\int_{X}\left[\dot{p}\ln\frac{p}{p_{\theta }}+\dot{p}-\frac{p}{p_{\theta }}{\dot{p}}_{\theta }\right]dx=\int_{X}\left[\dot{p}\ln\frac{p}{p_{\theta }}-\frac{p}{p_{\theta }}{\dot{p}}_{\theta }\right]dx.$$

Since

$$\frac{{\dot{p}}_{\theta }}{p_{\theta }}=\frac{d}{dt}\ln p_{\theta }=\dot{\theta }\cdot h-\partial_{\theta }\psi \cdot \dot{\theta }=\dot{\theta }\cdot \left(h-{\langle h\rangle }_{\theta }\right)$$

we have from (59)

$$\int_{X}p\frac{{\dot{p}}_{\theta }}{p_{\theta }}dx=\int_{X}\dot{\theta }\left(h-{\langle h\rangle }_{\theta }\right)dx=0$$

and recalling that *p* is a solution of FPE (51) we obtain

(65) $$\dot{b}=\frac{d}{dt}D\left(p|p_{\theta }\right)=\int_{X}\nabla \cdot \left(pD\nabla \ln\frac{p}{p_{\theta_{1}}}\right)\ln\frac{p}{p_{\theta }}dx=-\int_{X}pD\nabla \ln\frac{p}{p_{\theta_{1}}}\cdot \nabla \ln\frac{p}{p_{\theta }}dx$$

since we can get rid of the boundary term as done above.

The side $c=D(p_{\theta }|p_{\theta_{1}})$ does not contain the solution *p* of FPE therefore its time derivative can be computed as, see (45)

(66) $$\begin{array}{ccc} \dot{c} & = & \frac{d}{dt}D\left(p_{\theta }|p_{\theta_{1}}\right)=\int_{X}{\dot{p}}_{\theta }\ln\frac{p_{\theta }}{p_{\theta_{1}}}dx=\int_{X}p_{\theta }\frac{d}{dt}\left(\theta \cdot h-\psi \left(\theta \right)+k\right)\ln\frac{p_{\theta }}{p_{\theta_{1}}}dx\\ & = & \int_{X}p_{\theta }\dot{\theta }\left(h-{\langle h\rangle }_{\theta }\right)\left[\left(\theta -\theta_{1}\right)\cdot h+\psi \left(\theta_{1}\right)-\psi \left(\theta \right)\right]dx\\ & = & \langle h_{\alpha }h_{\beta }-\langle h_{\alpha }\rangle \langle h_{\beta }\rangle \rangle {\dot{\theta }}_{\alpha }{\left(\theta -\theta_{1}\right)}_{\beta }={\mathrm{cov}}_{\theta }\left(h_{\alpha },h_{\beta }\right){\dot{\theta }}_{\alpha }{\left(\theta -\theta_{1}\right)}_{\beta }\\ & = & g_{\alpha \beta }\left(\theta \right){\dot{\theta }}_{\alpha }{\left(\theta -\theta_{1}\right)}_{\beta }. \end{array}$$

On the other hand, $\dot{c}$ can also be computed from (64) and (65) as

(67) $$\begin{array}{ccc} \dot{c}=\dot{a}-\dot{b} & = & \int_{X}pD\nabla \ln\frac{p}{p_{\theta_{1}}}\cdot \nabla \left(\ln\frac{p}{p_{\theta }}-\ln\frac{p}{p_{\theta_{1}}}\right)dx\\ & = & \int_{X}pD\nabla \ln\frac{p}{p_{\theta_{1}}}\cdot \nabla \ln\frac{p_{\theta_{1}}}{p_{\theta }}\;dx\\ & = & -\int_{X}pD\nabla \ln\frac{p}{p_{\theta_{1}}}\cdot \nabla \ln\frac{p_{\theta }}{p_{\theta_{1}}}\;dx=V\left(p,\theta ,\theta_{1}\right). \end{array}$$

By equating (66) and (67) we obtain an ODE for the evolution of θ(t)

$$g_{\alpha \beta }\left(\theta \right){\dot{\theta }}_{\alpha }{\left(\theta -\theta_{1}\right)}_{\beta }=V\left(p,\theta ,\theta_{1}\right)$$

which depends on the unknown solution *p* of FPE. In the paper [3] the following approximation is made: if we substitute *p* with $p_{\theta }$ in $V(p,\theta ,\theta_{1})$ in (67) we get

(68) $$\begin{array}{ccc} V(p_{\theta },\theta ,\theta_{1}) & = & -\int_{X}p_{\theta }D\nabla \ln\frac{p_{\theta }}{p_{\theta_{1}}}\cdot \nabla \ln\frac{p_{\theta }}{p_{\theta_{1}}}dx\\ & = & -\int_{X}p_{\theta }D\nabla \left(h\cdot \left(\theta -\theta_{1}\right)\right)\cdot \nabla \left(h\cdot \left(\theta -\theta_{1}\right)\right)dx\\ & = & -\left(\int_{X}p_{\theta }d_{i}\partial_{i}h_{\alpha }\partial_{i}h_{\beta }dx\right){\left(\theta -\theta_{1}\right)}_{\alpha }{\left(\theta -\theta_{1}\right)}_{\beta }\\ & = & -{\langle B_{\alpha \beta }\rangle }_{\theta }{\left(\theta -\theta_{1}\right)}_{\alpha }{\left(\theta -\theta_{1}\right)}_{\beta } \end{array}$$

where we have introduced the the symmetric matrix

$$B_{\alpha \beta }={\left(D\nabla h\cdot h\right)}_{\alpha \beta }=d_{i}\partial_{i}h_{\alpha }\partial_{i}h_{\beta }.$$

By comparing (66) and (68) we obtain a closed form ODE for θ since $\theta -\theta_{1}\neq 0$

(69) $$A_{\alpha \beta }{\dot{\theta }}_{\alpha }=g_{\alpha \beta }\left(\theta \right){\dot{\theta }}_{\alpha }={\langle B_{\alpha \beta }\rangle }_{\theta }{\left(\theta_{1}-\theta \right)}_{\alpha }.$$

which is equation (5.2) in [3] or equation (12) in [4]. It can be given normal form since $g_{\alpha \beta }$ is invertible. In these paper the above equation is solved numerically and it is shown that it gives very good (sometimes surprisingly good) estimates of ${\langle h\rangle }_{p}$ using ${\langle h\rangle }_{\theta }$ even if θ is far from $\theta_{1}$.

Using the information geometry tools we have shown that the above triangular relation (62) holds independently from the assumption that θ be close to $\theta_{1}$ (called quasi equilibrium approximation in [2]). Moreover, is is evident from inspection of (65) that the substitution of *p* with $p_{\theta }$ renders $\dot{b}=0$ therefore $\dot{a}=\dot{c}$. Note that if $D_{ij}=d\delta_{ij}$ and we substitute *p* with $p_{\theta }$ in the above formula (67) we get

$$\dot{c}=\frac{d}{dt}D\left(p_{\theta }|p_{\theta_{1}}\right)=-dR\left(p_{\theta }|p_{\theta_{1}}\right).$$

Hence, if $p_{\theta_{1}}$ satisfies a LSI, we have exponential speed of convergence of $p_{\theta }$ to equilibrium distribution $p_{\theta_{1}}$, which explains the good behavior of the approximation.

### 6.2. A Dynamic Approximation Problem with External Parameters

We now suppose that the drift field (54) $D_{i}\left(x\right)=d\left(x_{i}\right)\partial_{i}\left(\theta \cdot h\left(x\right)\right)$ which defines the FPE depends on external parameters because h=h(x,u). We consider the same dynamic approximation problem of Section 6.1 with the extra degrees of freedoms given by the external parameters *u*. The approximation condition (57) now reads

$${\langle h\rangle }_{p}={\langle h\rangle }_{p_{z}}$$

where z=(θ,u). We take the time derivative of the above relation to find and ODE for *z*. We have

(70) $$\begin{array}{ccc} \frac{d}{dt}{\langle h\rangle }_{p_{z}} & = & \frac{d}{dt}\int_{X}hp_{z}dx={\langle \partial_{u}h\rangle }_{z}\dot{u}+\int_{X}hp_{z}\frac{d}{dt}\ln p_{z}dx\\ & = & {\langle \partial_{u}h\rangle }_{z}+\int_{X}hp_{z}\frac{d}{dt}\left(\theta \cdot h-\psi +k\right)dx\\ & = & {\langle \partial_{u}h\rangle }_{z}\dot{u}+\int_{X}p_{z}\left[h\left(h-\langle h\rangle \right)\dot{\theta }+\theta \cdot h\left(\partial_{u}h-\langle \partial_{u}h\rangle \right)\dot{u}\right]dx\\ & = & {\langle \partial_{u}h\rangle }_{z}\dot{u}+\omega \left(\dot{z}\right) \end{array}$$

and

$$\begin{array}{ccc} \frac{d}{dt}{\langle h\rangle }_{p} & = & \frac{d}{dt}\int_{X}hpdx={\langle \partial_{u}h\rangle }_{p}\dot{u}+\int_{X}h\partial_{t}pdx\\ & = & {\langle \partial_{u}h\rangle }_{p}\dot{u}+\int_{X}h\nabla \cdot \left(pD\nabla \ln\frac{p}{p_{\theta_{1}}}\right)dx\\ & = & {\langle \partial_{u}h\rangle }_{p}\dot{u}-\int_{X}pD\nabla \ln\frac{p}{p_{\theta_{1}}}\cdot \nabla h\;dx \end{array}$$

since the boundary term is vanishing. If we substitute *p* with $p_{z}$ in the last line we get

$$\begin{array}{ccc} \frac{d}{dt}{\langle h\rangle }_{p} & = & {\langle \partial_{u}h\rangle }_{p}\dot{u}-\int_{X}pD\nabla \ln\frac{p_{\theta }}{p_{\theta_{1}}}\cdot \nabla h\;dx\\ & = & {\langle \partial_{u}h\rangle }_{z}\dot{u}-{\langle B\rangle }_{z}\left(\theta -\theta_{1}\right) \end{array}$$

and therefore we have the ODE for *z* which is a direct generalization of (69)

(71) $$\omega \left(\dot{z}\right)=A\dot{\theta }+M\dot{u}={\langle B\rangle }_{z}\left(\theta_{1}-\theta \right).$$

Note that in this case we have considerably more freedom because we have a system of *a* ODEs for the d=a+b variables z=(θ,u) therefore we can assign the evolution u(t) of the external parameters to control the evolution θ(t).

## Appendix A. An Approximation Problem in Quantitative Genetics

We give a brief account of a classical problem in dynamics of quantitative traits whose approach to equilibrium described by a Fokker-Planck equation has been investigated in [2,3,4]. See also [37] for a gentle introduction to dynamics of populations. We consider a polygenic trait located in *n* biallelic loci (A,a). If we consider a sufficiently large population, the frequencies of genotype AA at locus *i* are described by *n* independent random variables $x=(x_{1},\ldots ,x_{n})$, $x_{i}\in \left[0,1\right]$. The dynamics of these allele frequencies can be described by a diffusion process under the action of stochastic forces which represent the effect of directional selection, dominance, mutation and random drift. This diffusion process is described by a linear Fokker-Planck equation whose equilibrium distribution is known from a long time ( see [38,39])

$$p\left(x\right)=e^{\theta \cdot h(x,u)-\psi (\theta ,u)+k(x)},\;x=\left(x_{1},\ldots ,x_{n}\right)\in X={\left[0,1\right]}^{n}$$

Below we show that it can be seen as a generalized exponential distribution with *natural* parameters $\theta =(\theta_{1},\theta_{2},\theta_{3},\theta_{4})$ and *external* parameters $u=\left(v,w\right)\in R_{+}^{2n}$. Setting $d\left(x_{i}\right)=x_{i}\left(1-x_{i}\right)$, we introduce the following four observables:

$$h_{1}\left(x,v\right)=-\sum_{i=1}^{n}v_{i}d^{\prime }\left(x_{i}\right)=-\sum_{i=1}^{n}v_{i}\left(2x_{i}-1\right)$$

$h_{1}$ is the directional selection and $v=(v_{1},\ldots ,v_{n})$ are external parameters describing the effects on loci;

$$h_{2}\left(x,w\right)=\sum_{i=1}^{n}w_{i}2d\left(x_{i}\right)=-\sum_{i=1}^{n}w_{i}2x_{i}\left(1-x_{i}\right)$$

$h_{2}$ is the dominance, $w=(w_{1},\ldots ,w_{n})$ are external parameters describing the effects on loci;

$$h_{3}\left(x\right)=\sum_{i=1}^{n}\ln x_{i}\;\mathrm{and}\;h_{4}\left(x\right)=\sum_{i=1}^{n}\ln\left(1-x_{i}\right)$$

$h_{3}$ describes forward and backward mutations and

$$k\left(x\right)=-\sum_{i=1}^{n}\ln x_{i}\left(1-x_{i}\right)$$

is the (non integrable) neutral distribution of allele frequencies in absence of selection and mutation. Since the random variables $x_{i}$ are independent, the free energy can be factorized using Fubini theorem

$$e^{\psi }=\int_{X}e^{\theta \cdot h+k}dx=\prod_{i=1}^{n}\int_{0}^{1}e^{-\theta_{1}v_{i}\left(2x_{i}-1\right)+\theta_{2}w_{i}2x_{i}\left(1-x_{i}\right)}x_{i}^{\theta_{3}-1}{\left(1-x_{i}\right)}^{\theta_{4}-1}dx_{i}.$$

We have $e^{\psi }<+\infty$ if $\theta_{3},\theta_{4}>0$ so θ=0 is a non feasible value. Moreover p(x) is integrable but tends to +∞ for *x* tending to ∂X if $1>\theta_{3},\theta_{4}>0$ while p=0 on ∂X if $\theta_{3},\theta_{4}>1$. Since the observables $h_{1}$ and $h_{2}$ have linear dependence on the external parameters *v* and *w*, this statistical model fails the injectivity condition 1. therefore it is not identifiable. The Fokker-Planck equation can be given the form (51) where $D=d\left(x_{i}\right)\delta_{ij}=x_{i}\left(1-x_{i}\right)$ is diagonal, but D=0 on ∂X therefore the inequalities like (52) that govern the trend to equilibrium are not strict ones due to the degeneracy of the diffusion matrix *D* on ∂X. This is a major source of difficulties in the analysis of this model as reported in [3]. A final remark is that the LSI sufficient condition Hess(V)≥σI becomes in this case (V=θ·h−ψ+k)

$$\partial_{i}\partial_{j}V=\partial_{i}\partial_{j}\left(\theta \cdot h-k\right)=4\theta_{2}w_{j}\delta_{ij}-\left(\frac{\theta_{4}}{{\left(1-x_{j}\right)}^{2}}-\frac{\theta_{3}}{x_{j}^{2}}\right)\delta_{ij}.$$

Therefore Hess(V) is not bounded below and the LSI condition fails. In the above cited papers the dynamic approximation procedure exposed in Section 6 is introduced to compute the evolution of the averages of the observables *h* without solving the FPE which is a computationally hard problem.

In this paper we have shown that this model problem is partly amenable to the controlled exponential family framework with some insight, however remain peculiar difficulties that prevent from a complete analysis of the model. We refer the interested reader to the specific literature, see [3].
